# Fucoidan Therapy for Extraintestinal Diseases: Targeting the Microbiota–Gut–Organ Axes

**DOI:** 10.3390/biom15121750

**Published:** 2025-12-18

**Authors:** Xian Sun, Ping Li, Baoduan Chen, Chong Chen, Jing Zhao, Sifan Sun

**Affiliations:** 1 School of Integrated Chinese and Western Medicine, Nanjing University of Chinese Medicine, Nanjing 210023, China; 290549@njucm.edu.cn; 2 No. 1 Clinical Medical College, Nanjing University of Chinese Medicine, Nanjing 210023, China; 20230052@njucm.edu.cn; 3 College of Pharmacy, Nanjing University of Chinese Medicine, Nanjing 210023, China; 4 Department of Nephrology, Affiliated Hospital of Nanjing University of Chinese Medicine, Nanjing 210029, China

**Keywords:** microbiota–gut–organ axes, fucoidan, extraintestinal diseases, fecal microbiota transplantation, strain supplementation

## Abstract

The microbiota–gut–organ axis is widely recognized as a pivotal mediator of systemic health, primarily through gut-derived immune, metabolic, and inflammatory signaling. Fucoidans, a class of fucose-containing sulfated polysaccharides predominantly composed of L-fucose and exclusively found in brown seaweeds, have been demonstrated to modulate gut microbiota composition and function, resulting in the enrichment of beneficial bacteria and the suppression of harmful species. They enhance the production of beneficial metabolites, such as short-chain fatty acids and specific bile acids, while suppressing harmful metabolites, including lipopolysaccharide, thereby ameliorating organ damage via key mechanisms such as the mitigation of oxidative stress and inhibition of inflammatory responses. Furthermore, fucoidan supplementation was found to restore intestinal barrier integrity. Using disease models including Parkinson’s disease, alcoholic liver disease, diabetic kidney disease, and obesity, the mechanisms through which fucoidans ameliorate extraintestinal diseases via the microbiota–gut–organ axis were elucidated. Microbiota-dependent mechanisms have been confirmed via experimental approaches such as fecal microbiota transplantation and specific bacterial strain supplementation. Fucoidans represent promising prebiotic agents for the restoration of microbial ecology and the treatment of extraintestinal diseases, highlighting the need for further clinical investigation.

## 1. Introduction

The gut microbiota plays an essential role that extends beyond digestion, critically influencing systemic health through a network of bidirectional communication pathways known as microbiota–gut–organ axes. These axes, including the gut–brain, gut–liver, gut–kidney, and gut–endocrine systems, facilitate complex interactions between the gut and extraintestinal organs via mechanisms such as signal transduction, generation of metabolic products, and modulation of inflammation and oxidative stress [1,2,3]. As the largest immune organ, the gut supports immune defense, maintains microbial ecological balance, and performs unique metabolic functions. Importantly, intestinal dysbiosis or impaired intestinal barrier function can lead to increased permeability and release of gut-derived toxins, such as trimethylamine N-oxide (TMAO), into circulation, thereby promoting metabolic, cardiovascular, and neurological disorders [4,5,6]. Conversely, microbial beneficial metabolites like short-chain fatty acids (SCFAs) and specific bile acids (BAs) help protect against multi-organ injuries [7,8,9]. Furthermore, dysfunction in peripheral organs can exacerbate gut damage, forming a vicious cycle that amplifies disease progression. Recent advances over the past five years have established the central role of microbiota–gut–organ axes disruption in the pathogenesis of inflammatory bowel disease (including Crohn’s disease) [10,11] and other extraintestinal disorders [12,13,14,15], highlighting the potential of targeting intestinal barrier restoration and microbial homeostasis as therapeutic strategies. However, despite growing interest, the regulatory mechanisms underlying these interactions remain incompletely elucidated, necessitating simplified experimental models to unravel this complexity and guide clinical interventions.

In recent decades, natural products have attracted considerable interest owing to their diverse pharmacological effects and unique biological activities [16,17,18,19]. However, many phytochemicals are hampered by poor bioavailability, resulting from limited solubility, rapid metabolism, and insufficient intestinal absorption. Despite these challenges, their intrinsic interaction with gut microbiota following oral administration has highlighted microbiota as a key mediator of their therapeutic mechanisms. Emerging nanotechnology-based delivery systems, such as lipid nanoparticles and polymeric nanocapsules, offer promising strategies to enhance bioavailability by protecting bioactive compounds from degradation and facilitating targeted delivery to microbial niches in the gut [20]. These complement accumulating evidence that natural compounds can modulate the composition and metabolic function of gut microbiota, thereby influencing metabolism, inflammation, oxidation, and systemic immunity, and ultimately altering the progression of extraintestinal diseases.

Among these natural compounds, fucoidan, a class of fucose-containing sulfated polysaccharides predominantly composed of L-fucose and exclusively found in brown seaweeds, has emerged as a promising candidate for treating disorders beyond the gut. These polysaccharides serve as major structural components in brown algae alongside alginate and cellulose and are notably absent in red and green algae [21]. Their structural features, including monosaccharide composition, sulfation pattern, branching degree, and molecular weight, vary considerably with algal species, growth conditions, harvesting season, and extraction method [22]. Critically, the biological functions of fucoidan are not solely attributable to its prebiotic-like properties; they are fundamentally determined by its structural features, such as molecular weight, sulfate content, and monosaccharide profile, which give rise to specific bioactive motifs [21]. These motifs, including distinct sulfation patterns and fucose-containing carbohydrate sequences, can function as biomimetic ligands, representing a mechanism of action beyond that of simple prebiotics. Fucoidan exhibits a broad spectrum of such bioactivities, including anti-inflammatory, antioxidant, immunomodulatory, anticancer, and antiviral effects. Notably, fucoidan resists digestion by gastric and pancreatic enzymes, reaching the colon intact, where it interacts extensively with gut microbiota. Through microbial fermentation, fucoidan modulates the abundance of beneficial bacteria and suppresses opportunistic pathogens, thereby promoting the production of bioactive metabolites, such as SCFAs and BAs. These microbiota-mediated mechanisms enable fucoidan to exert therapeutic effects across multiple organ systems via gut-derived axes. For instance, fucoidan alleviated HFD-induced obesity in mice through gut microbiota remodeling [23], and showed protective effects in ALD models by improving lipid metabolism and mitigating inflammation and oxidative stress via the gut-liver axis [24]. Furthermore, fucoidan ameliorated neuroinflammation and cognitive deficits in Alzheimer’s and Parkinson’s disease models, likely through gut–brain communication, by mitigating oxidative stress and apoptosis-related pathways [25,26]. Its renoprotective effects have also been linked to gut microbiota modulation [27,28]. Overall, fucoidan represents a paradigm for how natural polysaccharides can target extraintestinal diseases via microbiota–organ crosstalk. With its multifaceted mechanisms and high safety profile, fucoidan illustrates the broad potential of natural products in functional foods and precision medicine aimed at treating systemic diseases through microbiota–gut–organ pathways (Table 1).

However, a major challenge in current research on natural compounds lies in establishing causal links between microbiota–gut–organ axes modulation and the amelioration of extraintestinal diseases. Encouragingly, gut microbiota remodeling strategies, such as fecal microbiota transplantation (FMT) and defined strain supplementation, have provided both technical and empirical support to overcome this hurdle. These approaches serve as essential experimental tools for investigating how gut microbiota influence the therapeutic efficacy of natural compounds [38,39], including fucoidan, against extraintestinal pathologies. Microbial transfer techniques provide a means to elucidate the role of microbiota–gut–organ axes in mediating disease remission. In this review, fucoidan research in the context of Parkinson’s disease (PD) is emphasized to illustrate how FMT and specific microbial supplementation are utilized to verify fucoidan’s mechanistic pathways, particularly its neuroprotective effects mediated via the microbiota–gut–organ axes.

## 2. Microbiota–Gut–Organ Axes

### 2.1. Microbiota–Gut–Liver Axis

#### 2.1.1. Role of Natural Bioactive Compounds in the Microbiota–Gut–Liver Axis

The liver is highly vulnerable to bacterial translocation owing to its anatomical connection to the gut through the portal vein. This pathway facilitates the transit of bacteria, bacterial metabolites, and inflammatory mediators to the liver. Substantial evidence from experimental models supports the critical role of the microbiota–gut–liver axis in the pathogenesis of liver diseases, including nonalcoholic fatty liver disease (NAFLD), which is also referred to as metabolic dysfunction-associated steatotic liver disease (MASLD) [40,41,42], as well as alcoholic liver disease (ALD) [43], and liver cirrhosis [44,45,46]. Numerous natural products exhibit prebiotic or probiotic activities that collectively foster a favorable microbial environment, strengthen intestinal barrier function, and suppress pathogenic colonization, thereby reducing the hepatic influx of gut-derived injurious stimuli. These protective effects are mediated through multimodal mechanisms, such as modulation of microbiota and their metabolites, alleviation of oxidative stress, and inhibition of inflammatory pathways [47,48,49,50].

Natural bioactive compounds alleviate liver diseases by remodeling the gut microbiota, characterized by enhanced microbial diversity, expansion of beneficial commensals, and reduction in pathobionts. For example, the flavonoids quercetin and isoquercetin significantly modulated the gut microbial composition in NAFLD models: they markedly increased probiotics such as *Lactobacillus*, *Bifidobacterium*, *Akkermansia*, and *Dubosiella*, while suppressing pathogenic bacteria including *Clostridia*, *Klebsiella*, *Staphylococcus*, *Streptococcus*, *Flavonifractor*, and *Desulfovibrio* [51]. Similarly, in a mouse model of NASH, *Gynostemma pentaphyllum* extract (GPE) markedly reduced key pathological features, including hepatic inflammation, lipid accumulation, and peroxidation. This therapeutic benefit was partly attributed to a restructuring of the gut microbiota, characterized by an increase in beneficial *Akkermansia* and a reduction in opportunistic pathogens, including *Klebsiella*. The study demonstrated that the amelioration of liver disease by natural bioactive compounds, such as GPE, is partially mediated via microbiota remodeling. This process enhanced beneficial commensal bacteria while suppressing pathogenic pathobionts [52]. Furthermore, diammonium glycyrrhizinate (DG), a natural compound derived from licorice, effectively counteracted NAFLD by restoring gut ecosystem balance. It enriched microbial diversity, decreased the *Firmicutes*/*Bacteroidetes* ratio, and inhibited endotoxin-producing bacteria such as *Desulfovibrio*. Simultaneously, DG stimulated beneficial probiotics and SCFA-producing bacteria like *Ruminococcaceae*, leading to increased SCFA levels. These shifts supported intestinal barrier function through enhanced mucin secretion, strengthened tight junction (TJ), and reduced gut inflammation [53]. Additionally, emerging evidence demonstrates that natural bioactive compounds, including berberine (BBR), can precisely modulate the composition and production of microbial metabolites, which play a critical role in the progression of liver diseases. Specifically, BBR enhanced the secretion of microbial-derived SCFAs such as butyrate, contributing to improved hepatic lipid metabolism and attenuation of steatosis. This modulation of metabolite profiles mediated BBR’s ability to regulate gut hormone secretion, glucose homeostasis, and systemic energy balance, highlighting a microbiota-mediated mechanism that influenced liver health [54]. Similarly, previous studies revealed that *Apostichopus japonicus* fucoidan and *Scytosiphon lomentaria* fucoidan ameliorated ALD in murine models by selectively modulating microbial-derived SCFAs and BAs, respectively [55,56].

#### 2.1.2. Fucoidan and Hepatoprotection via the Gut–Liver Axis in ALD

Commensal depletion and pathobionts expansion in ALD: Chronic excessive alcohol intake severely affects the liver due to its central metabolic role, leading to ALD, the second most common cause of liver injury after viral hepatitis. The spectrum of ALD ranges from reversible steatosis and alcoholic steatohepatitis (ASH) to fibrosis, cirrhosis, and hepatocellular carcinoma (HCC) [57]. Although steatosis is highly prevalent among heavy drinkers, only 10–15% progress to cirrhosis, whereas ASH develops in 20–40% of cases. Alcohol-related cirrhosis accounts for approximately 50% of all cirrhosis-associated mortality, significantly contributing to the global annual total of two million liver-related deaths [58]. Discontinuation of alcohol use constitutes the most effective intervention to prevent disease advancement. Given the inadequate efficacy and adverse effects of current therapies, elucidating the genetic and environmental determinants of ALD and developing novel treatment strategies are urgently required.

Emerging evidence indicates that alcohol consumption induces gut microbial dysbiosis, which disrupts the gut–liver axis and contributes to the pathogenesis of ALD. Although only a minority of heavy drinkers progress to advanced ALD due to factors such as genetics, drinking patterns, and comorbidities, the gut microbiota has been identified as a critical modifier of disease susceptibility and severity. Clinical observations revealed consistent alterations in the gut microbiota of ALD patients, characterized by reduced bacterial diversity, depletion of commensal bacteria such as *Lachnospiraceae* and *Ruminococcaceae*, which produced beneficial SCFAs, and enrichment of potential pathobionts including *Enterobacteriaceae*, *Streptococcaceae*, and *Enterococcus* [59]. Functional evidence from FMT studies demonstrated that GF mice colonized with microbiota from alcoholic hepatitis patients developed more severe liver inflammation and increased intestinal permeability following alcohol feeding than those receiving microbiota from healthy donors. Conversely, subsequent transplantation of healthy microbiota attenuated alcohol-induced liver injury despite ongoing exposure [29]. These microbial shifts impaired intestinal barrier integrity, leading to increased translocation of bacteria and microbial products to the liver via the portal vein, thereby exacerbating hepatic inflammation and injury [60]. Thus, targeting gut microbiota dysbiosis represents a promising therapeutic strategy for ALD.

SCFA and BA modulation in the gut-liver axis: Specific mechanistic elucidation based on previous studies. (1) Regulation of commensals and pathobionts: Studies have demonstrated that fucoidan derived from diverse marine sources modulated gut microbiota composition by promoting beneficial commensals and suppressing pathogenic taxa, thereby ameliorating ALD. Sun et al. reported that *Scytosiphon lomentaria* fucoidan increased microbial diversity, elevated beneficial genera such as *Parabacteroides*, *Bacteroides*, and *Faecalibaculum*, and reduced *Proteobacteria* and *Bacteroidetes* [24]. Similarly, Li et al. showed that *Apostichopus japonicus* fucoidan decreased the pro-inflammatory bacterium (*Clostridia_UCG-014*), while enriching beneficial families *Muribaculaceae* and *Lactobacillaceae*, which produce anti-inflammatory metabolites including butyrate and indole-3-propionic acid [55]. Further supporting these findings, Wang et al. identified *Parabacteroides distasonis* as a key mediator through which *Scytosiphon lomentaria* fucoidan exerted hepatoprotective effects via the gut–liver axis [56]. Collectively, these studies indicated that fucoidan mitigated ALD by selectively enhancing commensal microbiota and inhibiting pathobionts.

(2) Modulation of SCFA and BA: Chronic alcohol consumption compromises intestinal barrier function through direct impairment of TJ and induction of gut microbial dysbiosis, notably depleting commensals that produce SCFAs. Families such as *Lachnospiraceae* and *Ruminococcaceae*, which are major producers of SCFAs, are consistently diminished in patients with ALD [59]. This reduction leads to decreased levels of key SCFAs, such as acetate, propionate, and butyrate, which are critical for maintaining intestinal homeostasis. The resulting deficiency in SCFAs disrupts epithelial energy metabolism, weakens intercellular junctions, diminishes the mucous layer, and suppresses antimicrobial peptide production. Collectively, these impairments lead to increased intestinal permeability, thereby promoting the translocation of microbial products into the systemic circulation [60]. SCFAs also act as signaling molecules modulating hepatic protective pathways such as AMPK and Nrf-2 via the gut–liver axis, thereby attenuating inflammation and hepatic steatosis [61]. A study by Li et al. provided mechanistic insight into the role of SCFAs in restoring barrier integrity and mitigating ALD. In rodent models, alcohol exposure was demonstrated to significantly reduce the levels of multiple SCFAs, including acetate, propionate, butyrate, isobutyrate, valerate, and isovalerate. Treatment with *Apostichopus japonicus* fucoidan notably reversed these deficits, particularly restoring acetate and butyrate concentrations. Importantly, butyrate was shown to upregulate TJ protein zonula occludens-1 (Zo-1) expression and reduce LPS translocation, thereby ameliorating alcohol-induced liver injury. These findings indicated that fucoidan ameliorated intestinal hyperpermeability and liver damage primarily by enhancing SCFA production, thereby highlighting the therapeutic potential of SCFA-mediated barrier restoration in ALD [55].

The gut–liver axis represents a well-established bidirectional communication system in which luminal components, delivered via the portal circulation, influence liver physiology and disease, while hepatically derived molecules such as BAs shape the intestinal environment through biliary secretion. BAs are synthesized in the liver from cholesterol, conjugated primarily with glycine or taurine, and secreted into the intestine, where they facilitate lipid absorption and play a critical role in maintaining intestinal homeostasis [62]. Approximately 95% of primary BAs are reabsorbed in the ileum and recycled via enterohepatic circulation; the remaining 5% are metabolized by gut microbiota into secondary BAs [30]. This microbial transformation not only modulates BA composition but also activates key signaling pathways, including the farnesoid X receptor (FXR), which regulates BA synthesis, hepatic inflammation, and gut barrier integrity through the induction of antimicrobial peptides [63,64,65]. Disruption of gut microbiota can alter BA metabolism by enhancing secondary BA conversion and reducing primary BA reabsorption, thereby impairing FXR signaling and promoting dysbiosis and liver injury. Recent studies highlight the therapeutic potential of modulating BA metabolism in ALD. Sun et al. reported that *Scytosiphon lomentaria* fucoidan administration in an ALD model significantly reduced levels of specific BAs, including tauro-β-muricholic acid (T-MCA), TUDCA, glycolithocholic acid (GLCA), and UDCA, while increasing LCA and DCA, suggesting a normalization of BA pool composition accompanied by hepatoprotective effects [24]. Complementing these findings, Wang et al. demonstrated that *Scytosiphon lomentaria* fucoidan attenuated alcohol-induced liver injury by enriching *Parabacteroides distasonis*, a bacterial strain capable of modulating BA metabolism. *Parabacteroides distasonis* colonization reduced total BA levels and improved BA composition, subsequently regulating the expression of key genes involved in BA synthesis, transport, and reabsorption in the liver and ileum [56]. Together, these studies indicated that fucoidan partially ameliorated ALD through microbial–BA crosstalk, restoring enterohepatic signaling and highlighting the potential of microbiota-targeted therapies in managing hepatobiliary disorders.

Mechanistic insights: Fucoidans derived from marine sources, such as *Scytosiphon lomentaria* and *Apostichopus japonicus*, exhibit protective effects against alcohol-induced liver injury through multimodal regulation of the gut-liver axis. Both *Scytosiphon lomentaria* fucoidan and *Apostichopus japonicus* fucoidan ameliorated ALD by modulating gut microbiota composition, enhancing microbial metabolites, and reinforcing intestinal barrier integrity. Specifically, *Apostichopus japonicus* fucoidan administration reduced serum total cholesterol and LDL-C levels, increased HDL-C, and restored hepatic antioxidant capacity by elevating glutathione levels. Furthermore, *Apostichopus japonicus* fucoidan upregulated TJ proteins Zo-1 and occludin, thereby repairing the intestinal barrier and promoting SCFA production. *Scytosiphon lomentaria* fucoidan exerted its hepatoprotective effects largely through enrichment of *Parabacteroides distasonis*, a key commensal bacterium involved in BA metabolism and anti-inflammatory signaling. *Parabacteroides distasonis* modulation was associated with inhibition of NF-κB and MAPK pathways and activation of the Nrf2 antioxidant pathway, leading to reduced hepatic inflammation and oxidative stress. Additionally, *Scytosiphon lomentaria* fucoidan contributed to beneficial alterations in microbial diversity, amino acid metabolism, and BA profiles [24,55,56].

Despite promising results, several challenges require further investigation. These include establishing dose–response and structure–function relationships for different fucoidans, improving accuracy in BA detection and microbial quantification, clarifying the specific role of *Parabacteroides distasonis* in BA modulation, and elucidating the precise mechanisms underlying fucoidan-mediated microbiota-host interactions. Addressing these questions will strengthen the potential application of fucoidans as prebiotic agents for the management of liver diseases.

### 2.2. Microbiota–Gut–Kidney Axis

#### 2.2.1. Role of Natural Bioactive Compounds in the Microbiota–Gut–Kidney Axis

The microbiota–gut–kidney axis provides a comprehensive framework for understanding the bidirectional communication between gut microbiota and renal pathophysiology [66,67,68,69]. Emerging evidence highlights the significant role of this axis in the pathogenesis of kidney disease, wherein microbial metabolites, including TMAO, SCFAs, and BAs, contribute to disease development and progression [70,71]. Furthermore, a compromised intestinal barrier facilitates the translocation of toxins into the systemic circulation, thereby aggravating renal injury [72,73,74]. Natural bioactive compounds have shown therapeutic potential by targeting the microbiota–gut–kidney axis, with mechanisms that include restoration of beneficial microbiota, suppression of pathogenic bacteria, regulation of microbial metabolites, and repair of intestinal barrier integrity.

Taking natural bioactive polysaccharides as a representative example, Dong et al. demonstrated that corn silk polysaccharides (CSPs), as natural bioactive compounds, exerted therapeutic effects on diabetic kidney disease (DKD) by modulating the gut-kidney axis. Their study revealed that CSPs significantly altered specific metabolic pathways, including glycerophosphate, fatty acid, BA, tyrosine, tryptophan, and phenylalanine metabolism, and regulated gut microbiota such as *Firmicutes*, *Bacteroides*, *Lachnospiraceae_NK4A136_group*, and *Dubosiella*. These findings suggested that the renoprotective mechanism of CSPs was associated with the restoration of gut microbial ecology and subsequent regulation of microbial metabolite profiles. Furthermore, Zhang et al. demonstrated that the polysaccharide from Moutan Cortex (MC-Pa), a natural bioactive compound, ameliorated DKD through modulation of the microbiota–gut-–kidney axis. Their findings indicated that MC-Pa alleviated hyperglycemia and renal injury, while concurrently restoring gut microbiota composition, enhancing intestinal barrier function, elevating SCFA levels, and suppressing systemic inflammation. Moreover, *Saccharina japonica* fucoidan demonstrated a protective effect against renal injury, potentially through the maintenance of intestinal homeostasis. Specifically, fucoidan administration reduced serum LPS levels, enhanced intestinal mucosal barrier function, and markedly shifted gut microbiota composition by decreasing the abundances of *Blautia*, *Muribaculaceae*, and *Dubosiella*, while promoting *Lactobacillus* growth. Furthermore, high-dose fucoidan supplementation significantly increased butyric acid content, suggesting a metabolite-mediated mechanism contributing to its renoprotective efficacy [28]. Similarly, Zhong et al. reported that *Undaria pinnatifida* fucoidan effectively improved glomerular hyperfiltration and renal fibrosis in a DKD model. This therapeutic outcome was associated with a significant enrichment of SCFA-producing gut bacteria and an elevated concentration of acetic acid in the cecum [27]. These findings further support the concept that natural bioactive compounds, including fucoidan, target the microbiota–gut–kidney axis by restoring beneficial microbiota and associated metabolic pathways.

#### 2.2.2. Fucoidan and Renal Protection via Gut–Kidney Axis in DKD

Gut microbiota disruption in DKD: DKD, a common microvascular complication of diabetes mellitus (DM), is characterized by progressive renal damage, proteinuria, hypertension, and edema. It represents a major cause of end-stage renal disease (ESRD), affecting approximately 20–40% of patients with DM [75], one-third of whom will progress to ESRD [76]. Individuals with DKD face a mortality rate 30 times higher than diabetic patients without kidney involvement [77], contributing substantially to healthcare economic burdens. Current management strategies focus on lifestyle modification, metabolic control, and antihypertensive and hypoglycemic agents to slow disease progression and improve quality of life [78]. However, the clinical management of DKD remains limited due to its complex pathogenesis, further underscoring the need for novel therapeutic strategies. Growing evidence now reveals a bidirectional microbiota–kidney crosstalk that becomes particularly pronounced during progressive renal dysfunction, highlighting its potential as a novel therapeutic target.

Accumulating evidence indicates that DKD is associated with significant gut microbiota dysbiosis, characterized by altered microbial composition and diversity. Compared to healthy controls, patients with DKD exhibited reduced bacterial richness and distinct beta diversity [79]. A consistent pattern emerges from clinical and preclinical studies indicating that DKD is marked by a decline in beneficial SCFA-producing bacteria and an expansion of pathobionts [80,81]. Specifically, clinical studies, including a systematic review and meta-analysis that encompassed 16 studies with 578 DKD patients and 444 healthy controls, reported a decreased abundance of the phylum *Firmicutes*, as well as families such as *Lachnospiraceae*, and genera including *Roseburia*, *Prevotella*, and *Bifidobacterium*. Conversely, DKD patients exhibited an enrichment of *Proteobacteria*, *Actinobacteria*, *Enterobacteriaceae*, and genera such as *Enterococcus*, *Escherichia*, *Klebsiella*, and *Akkermansia*, with species such as *Escherichia coli* (*E. coli)* being particularly prominent [82]. These findings were corroborated in rodent models of DKD, which similarly exhibited reduced alpha diversity and depletion of SCFA-producing genera such as *Ruminococcus*, *Rikenella*, and *Bacteroides*, including *Bacteroides acidifaciens* (*B. acidifaciens*) [83]. Collectively, human and animal data demonstrated that DKD was closely linked to gut ecological disruption, manifested as a loss of microbial diversity, reduction in commensal beneficial taxa, and proliferation of pathogenic organisms, contributing to disease progression and highlighting the gut microbiota as a potential therapeutic target.

SCFA and BA pathways in gut–kidney axis regulation: Emerging evidence has elucidated the critical roles of gut microbial metabolites in the pathogenesis of DKD. Elevated circulating levels of TMAO contribute directly to renal dysfunction by promoting inflammation, oxidative stress, and fibrosis, as demonstrated in patients and animal models with CKD [84,85,86]. In contrast, SCFAs and specific BAs exhibit protective effects. Reductions in SCFA-producing bacteria and lower SCFA levels correlate with kidney disease [87], whereas butyrate and acetate improve barrier integrity and mitigate renal damage through G-protein-coupled receptors (GPCRs) and histone deacetylase (HDAC) inhibition [88,89]. Similarly, a previous study demonstrated that the addition of TUDCA to conventional telmisartan therapy markedly enhanced the restoration of renal function and suppression of apoptotic and fibrotic signaling in a DKD model [90]. Complementing these findings, Zhong et al. demonstrated that *Undaria pinnatifida* fucoidan modulated gut microbiota by enriching SCFA-producing bacteria, particularly *Streptococcus* and *Lactobacillaceae*, resulting in significantly elevated cecal concentrations of acetate and total SCFAs. Given that acetate ameliorates mitochondrial dysfunction and tubular inflammation, Zhong’s work provided a mechanistic basis for interventions aimed at restoring microbial SCFA production as a therapeutic strategy in DKD [27]. Nevertheless, the precise regulation of TMAO and the potential interplay between SCFAs, BAs, and TMAO in DKD progression warrant further investigation.

Mechanistic insights: *Undaria pinnatifida* fucoidan significantly ameliorated renal hyperfiltration and fibrosis in early-stage DKD. The renoprotective effects of *Undaria pinnatifida* fucoidan were mediated through multiple interconnected mechanisms. *Undaria pinnatifida* fucoidan enriched SCFAs-producing bacteria and increased acetic acid concentration in the cecum, thereby enhancing renal ATP production and ameliorating mitochondrial dysfunction. Subsequently, *Undaria pinnatifida* fucoidan attenuated renal inflammation and fibrosis by inhibiting the MAPKs signaling pathway, which was known to play a crucial role in the progression of kidney diseases. These findings demonstrated that *Undaria pinnatifida* fucoidan improved diet-induced renal dysfunction by modulating the microbiota–gut–kidney axis, enhancing mitochondrial bioenergetics, and suppressing MAPKs-mediated inflammatory and fibrotic responses. This study highlights the potential of fucoidan as a multifaceted therapeutic agent for mitigating early-stage DKD [27].

**Figure 1:**
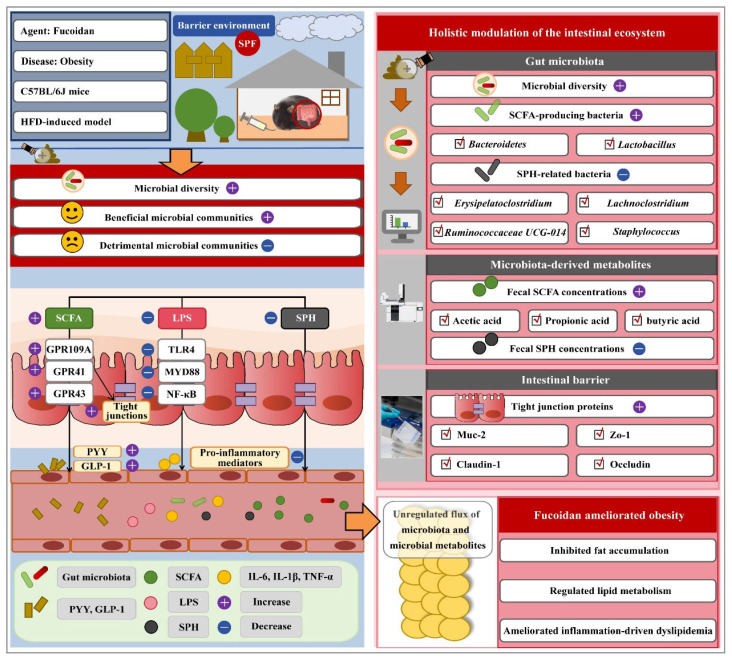
Mechanism schematic of fucoidan modulating obesity through the gut–endocrine axis.

Additionally, emerging evidence indicated that fucoidans derived from diverse seaweed species ameliorated DM and obesity by modulating the gut-endocrine axis. For instance, Wu et al. demonstrated that *Sargassum fusiforme* fucoidan reduced hyperglycemia, serum lipids, food intake, and oxidative stress in HFD/STZ-induced diabetic mice, alongside modulating gut microbiota and elevating colonic carnitine and choline levels [91]. Similarly, Zhang et al. reported that *Saccharina japonica* fucoidan improved glucose and lipid metabolism, oxidative stress, and pancreatic islet integrity in type 2 diabetic models, correlating with increased *Lactobacillus* and *Allobaculum* abundance and altered microbial metabolic pathways [92]. The anti-obesity effect of this fucoidan was baseline microbiota-dependent, attenuating obesity only in penicillin-treated mice with a favorable microbial profile, independent of SCFA production [23]. Zuo et al. further showed that *Sargassum fusiforme* fucoidan counteracted diet-induced obesity by enhancing energy expenditure via activation of brown adipose tissue and browning of inguinal white adipose tissue; these effects were abolished by antibiotic treatment, underscoring the essential role of gut microbiota [93]. Notably, Lin et al. revealed that *Saccharina japonica* fucoidan ameliorated metabolic dysfunction via multi-targeted actions on the gut–organ axis (Figure 1). Its administration reshaped gut microbiota, increasing *Bacteroidetes* and *Lactobacillus*, elevated microbial metabolites including SCFAs (acetate, propionate, butyrate), and modulated sphingosine metabolism. These changes enhanced intestinal barrier integrity through upregulation of TJ proteins (Zo-1, Claudin-1, Occludin), reducing microbial translocation. Concurrently, systemic inflammation was suppressed via downregulation of pro-inflammatory cytokines (TNF-α, IL-6, IL-1β, Mcp-1). Ultimately, fat accumulation was reduced through upregulation of thermogenic genes (*Ucp-1*, *Prdm16*, *Pgc-1α*) and fatty acid oxidation genes (*Ppar-α*, *Ppar-γ*, *Cpt-1*), and suppression of lipogenic pathways [94].

### 2.3. Microbiota–Gut–Brain Axis

The microbiota–gut–brain axis represents a crucial therapeutic target for neurodegenerative disorders [95,96,97]. Among various interventions, fucoidan has demonstrated significant potential in modulating this axis to ameliorate PD pathology. Mechanistic studies revealed that fucoidan exerted its beneficial effects through three complementary pathways: gut microbiota remodeling, reduction in circulating LPS levels, and restoration of intestinal barrier integrity. This section critically evaluated the evidence supporting fucoidan-mediated neuroprotection via these coordinated mechanisms along the microbiota–gut–brain axis in PD.

#### 2.3.1. Gut Microbiota Dysbiosis in PD

PD represents the second most prevalent neurodegenerative disorder globally after AD and is the fastest-growing neurological condition. Primarily affecting individuals aged ≥60 years, with an estimated global prevalence of 6–8 million cases (approximately 200 per 100,000 people) [98,99]. Driven by aging populations, increased longevity, and environmental factors, the global burden of PD is projected to double by 2040 [100]. The disease clinically manifests with characteristic motor impairments, including resting tremor, bradykinesia, rigidity, and postural instability with gait impairment. These motor deficits predominantly result from the progressive degeneration of dopaminergic neurons within the substantia nigra pars compacta, disrupting nigrostriatal pathway function. This neuronal loss leads to a critical striatal dopamine deficiency, thereby disturbing the balance between inhibitory dopaminergic and excitatory cholinergic neurotransmission [101]. Pathologically, PD is defined by the loss of nigral dopaminergic neurons and abnormal intracellular aggregation of α-synuclein protein. Crucially, PD also encompasses significant non-motor manifestations, including constipation, anxiety, depression, cognitive alterations, and gastrointestinal dysfunction (potentially linked to gut dysbiosis) [102], which severely impact quality of life. While genetic factors contribute to approximately 10% of cases, PD is fundamentally a chronic, progressive movement disorder with a substantial and worsening impact on mobility and muscle control over time.

Accumulating clinical and experimental evidence establishes gut microbiota dysbiosis in PD patients and animal models, highlighting a crucial role for the microbiota–gut–brain axis in disease pathogenesis. This bidirectional communication network modulates brain health through microbial metabolite production, barrier integrity maintenance, vagus nerve signaling, neuroendocrine pathways, and immune interactions [103]. Disrupted gut microbiota homeostasis represents a potential PD risk factor. Comprehensive analyses, including large-scale 16S rRNA gene sequencing studies and meta-analyses of case–control cohorts, consistently demonstrated significant microbial shifts in geographically diverse PD populations. These shifts were characterized by a depletion in the abundance of SCFA-producing taxa (*Prevotellaceae*, *Faecalibacterium*, *Lachnospiraceae*), concurrent with an enrichment of *Bifidobacterium*, *Lactobacillus*, *Akkermansia*, *Ruminococcaceae*, *Christensenellaceae*, and various pathogenic microbiota (e.g., *Streptococcus*, *Enterococcus*, *Parabacteroides*, *Enterobacteriaceae* members). Such dysbiosis significantly impaired microbiota–gut–brain axis function [104,105]. Preclinical evidence confirmed causality: FMT from PD-model animals induced PD-like pathology and motor deficits in recipients, whereas germ-free (GF) α-synuclein-overexpressing mice exhibited attenuated symptomatology [106,107]. Notably, in rotenone (ROT)-induced PD models, altered microbial metabolites and intestinal dysbiosis precede motor dysfunction, identifying gut dysbiosis as an early driver of central neuropathology and a validated PD risk factor [108].

#### 2.3.2. Fucoidan and Neuroprotection via the Gut–Brain Axis in PD

Specific mechanistic elucidation based on a study by Yang et al. [25]: (1) Modulation of *L. murinus*, *L. johnsonii*, and *A. muciniphila*: Gut microbiota composition significantly diverges in PD patients compared to healthy individuals, with *Lactobacillaceae* consistently exhibiting elevated abundance. Paradoxically, this family exerts dual effects in PD pathogenesis. While gut-colonizing *Lactobacillaceae* species are associated with aggravated motor deficits and premature levodopa degradation, probiotic formulations containing *Lactobacillus* correlate with improved motor function and alleviated gastrointestinal symptoms [109]. Supporting species-specific roles, Yang et al. demonstrated that *Saccharina japonica* fucoidan attenuated ROT-induced Parkinsonism in mice, revealing a critical divergence: a significant reduction in pro-inflammatory *L. johnsonii* concurrent with increased abundance of neuroprotective *L. murinu* [25]. Mechanistically, *L. johnsonii* upregulated pro-inflammatory cytokines (IL-12, IFN-γ, TNF-α, IL-1β, IL-6) in vitro [110], while fucoidan-induced *L. murinus* enhanced intestinal barrier integrity and reduced systemic inflammation by activating TLR2 on M2 macrophages to induce IL-10 release [111]. These differential shifts provide a mechanistic basis for prior conflicting clinical observations and highlight the therapeutic potential of precision microbiota modulation. Moreover, fucoidan treatment led to a significant reduction in *A. muciniphila* abundance. Gut commensals dynamically regulate neurological disorders via bidirectional gut–brain axis communication. *A. muciniphila*, a well-documented commensal bacterium, exhibits systemic benefits through enhanced immunological and metabolic functions, positioning it as a promising probiotic. Paradoxically, its role in neuropsychiatric disorders remains contentious [112]. Previous studies detected significantly reduced fecal abundance of *A. muciniphila* in PD rat models; however, exogenous administration of this bacterium ameliorated 6-OHDA-induced motor dysfunction, preserved dopaminergic neuronal integrity, and attenuated neuroinflammatory mediator release [113]. Conversely, clinical observations reported significantly elevated *A. muciniphila* abundance in PD patients [114,115]; its mucin-degrading capacity may compromise intestinal barrier function, increase permeability, and expose the enteric plexus to pro-inflammatory toxins (e.g., LPS), mechanisms suggested to potentially drive PD progression. We propose that these context-dependent actions reflect *A. muciniphila*’s integration within the complex gut ecosystem, where functional outcomes are determined by microbial community dynamics and relative species abundance. Thus, the mechanistic contribution of *A. muciniphila* to PD development warrants further investigation.

(2) Attenuation of LPS levels: Mounting evidence demonstrates the critical role of gut microbial metabolites in PD pathogenesis, primarily mediated through the microbiota–gut–brain axis. Key microbial metabolites implicated include SCFAs, trimethylamine-N-oxide (TMAO), BAs, and LPS. SCFAs, TMAO, and BAs are small molecules produced from dietary substrate metabolism or host molecule modification by gut bacteria; they significantly influence intestinal barrier integrity, energy homeostasis, and immune function. Crucially, dysregulation of these metabolites, alongside LPS, a potent pro-inflammatory bacterial component, is increasingly linked to PD. Metabolomics advancements have been instrumental in identifying specific disruptions, including altered SCFA profiles, BA metabolism, and elevated TMAO and LPS levels, as key contributors to PD pathology.

SCFAs, primarily acetate, propionate, and butyrate, are organic compounds produced through the fermentation of dietary fiber by gut microbiota. These metabolites serve critical physiological roles: maintaining intestinal barrier integrity, modulating immune homeostasis, regulating microglial maturation in the central nervous system (CNS), and acting as energy substrates for colonocytes [116]. Emerging evidence links disrupted SCFA homeostasis to PD pathogenesis. PD patients exhibited significantly reduced fecal SCFA levels and diminished abundances of SCFA-producing bacteria. This deficiency compromised intestinal barrier function and blood–brain barrier (BBB) integrity, facilitating translocation of endotoxins and neurotoxins while promoting pathological α-synuclein aggregation and neuroinflammation [117]. Specifically, low SCFA levels correlated with poor cognitive function and worsened postural instability in PD [118,119]. Mechanistically, SCFA depletion dysregulated immune responses by downregulating regulatory T cells and upregulating T-helper 17 cells, exacerbating neuronal inflammation and neurodegeneration [119]. Butyrate, a key SCFA, demonstrated neuroprotective effects in PD models: it attenuated dopaminergic neuron loss, reduced α-synuclein aggregation via autophagy induction (dependent on Atg5 and PI3K/Akt/mTOR pathways) [120]. Furthermore, butyrate could remedy motor symptoms and pathological alterations in PD mice by restoring the intestinal barrier with activated GPR109A [121]. Paradoxically, propionate may be associated with poorer motor scores (UPDRS-III), highlighting SCFA-specific effects [122]. TMAO is a gut microbiota-derived metabolite generated through a pathway involving diet and the host-microbiota interplay: gut microbes convert dietary choline, carnitine, and phosphatidylcholine into trimethylamine (TMA), which is subsequently oxidized in the liver by flavin-containing monooxygenases (primarily FMO3) [123,124,125]. Clinical metabolomic studies reported elevated circulating TMAO levels in PD patients, though these elevations appear independent of disease characteristics, treatment status, and lifestyle [126]. Notably, conflicting evidence exists regarding TMAO alterations in PD, with studies reporting either increased or decreased plasma/stool levels [127,128,129]. TMAO contributed to neurodegeneration through multiple mechanisms. For instance, it disrupted BBB integrity, which facilitated brain entry of pathogenic substances and amplified microglial activation and neuronal damage. In vitro and in vivo studies demonstrated that TMAO activated pro-inflammatory pathways, notably NF-κB and the NLRP3 inflammasome, leading to increased production of cytokines (e.g., IL-1β, IL-18, TNF-α) and exacerbating neuroinflammation and oxidative stress [130,131,132]. Significantly, Hoyles L et al. demonstrated that physiological concentrations of the dietary TMAO enhanced blood–brain barrier (BBB) integrity and conferred protection against inflammatory insult; this protective effect was mediated by the TJ regulator annexin A1. In contrast, the TMAO precursor trimethylamine (TMA) impaired BBB function and disrupted TJ integrity [133]. In PD patients, the gut microbiota-mediated biotransformation of primary to secondary BAs was altered, leading to elevated cecal levels of deoxycholic acid (DCA) and lithocholic acid (LCA) [134]. Conversely, specific BAs demonstrated significant neuroprotective potential. Tauroursodeoxycholic acid (TUDCA) has shown important anti-apoptotic and neuroprotective activities, with numerous experimental and clinical evidence suggesting its possible therapeutic use as a disease-modifier in neurodegenerative diseases. In rodent PD models, TUDCA (50 mg/kg) demonstrated neuroprotective efficacy by mitigating ROS-mediated apoptosis and preventing MPTP-induced neurodegeneration. Its mechanism involved suppressing JNK phosphorylation, reducing ROS production, enhancing glutathione S-transferase (GST) activity, and activating Akt signaling pathways [135,136]. Similarly, ursodeoxycholic acid (UDCA) exhibited neuroprotection in both human studies and rodent PD models [137,138,139]. Notably, BAs enter the brain primarily via systemic circulation through diffusion or active transport by BA transporters, rather than through local synthesis in astrocytes and neurons. Although astrocytes and neurons express BA-producing enzymes, brain concentrations mirror circulating levels [140]. Collectively, these findings position the modulation of gut-derived BAs as a compelling therapeutic strategy for PD.

Yang et al. demonstrated that *Saccharina japonica* fucoidan ameliorated PD by restoring gut microbial metabolites with specific targeting of LPS. LPS, an endotoxin derived from Gram-negative gut bacteria and a significant cell wall constituent, serves as a critical biomarker of bacterial translocation and gut barrier dysfunction in PD [31,32,141]. The gut microbiota represents a primary source of LPS, a highly immunogenic molecule. In PD, gut dysbiosis compromises the intestinal barrier, facilitating bacterial translocation and elevating systemic LPS levels [142]. LPS exerts potent immunogenic effects primarily by binding TLR4, the main receptor for LPS and a key mediator in gut and brain homeostasis. This binding activates downstream signaling pathways, notably TLR4/NF-κB, driving the production of pro-inflammatory cytokines, including TNF-α and IL-1β [143]. This cascade triggers a cycle of intestinal inflammation and oxidative stress, driving increased permeability, disruption of epithelial TJ, and early intestinal dysfunction, which collectively exacerbate the entry of LPS into the bloodstream [144]. Critically, circulating LPS propagates inflammation via the blood and gut–brain axis, amplifying central inflammation, damaging nigrostriatal dopamine neurons, influencing α-synuclein pathology, and contributing significantly to PD pathogenesis and neurodegeneration [97]. Notably, Yang et al. demonstrated that *Saccharina japonica* fucoidan treatment in ROT-induced PD mice significantly reduced serum levels of LPS, TNF-α, and IL-1β, and decreased the expression of TLR4 and NF-κB in key PD-affected regions (substantia nigra, striatum, and colon) [25]. These findings indicated that fucoidan exerted its anti-peripheral and central inflammatory effects by modulating the gut microbiota to suppress LPS production and concurrently inhibiting the LPS/TLR4/NF-κB signaling pathway, suggesting a promising therapeutic approach targeting gut-derived inflammation and its transmission along the gut–brain axis in PD.

(3) Impaired intestinal barrier: The intestinal barrier is a fundamental structure in health and disease, consisting of three coordinated layers: the mucus layer, the epithelial barrier, and the gut vascular barrier. As the largest interface between the host and the external environment, this multilayered system regulates the selective absorption of nutrients, electrolytes, and water while preventing the translocation of pathogens and harmful luminal substances [145]. A single layer of epithelial cells interconnected by TJ proteins, such as Zo-1, Claudin-1, and occludin, forms the core of the epithelial barrier and controls paracellular permeability [146]. Underlying this, the gut vascular barrier, which is composed of endothelial cells with tight and adherens junctions along with pericytes and enteric glia, further restricts the systemic dissemination of microbial products and toxins [147,148]. Impairment of this integrated barrier, often described as leaky gut, has been strongly implicated in PD [149,150]. Patients with PD exhibited decreased expression of key TJ proteins, including ZO-1 [151], Occludin [152], and Claudin-1 [153], a dysfunction that facilitates the translocation of gut-derived pathogens and pro-inflammatory mediators such as LPS into systemic circulation. This phenomenon is supported by elevated circulating LPS and reduced LPS-binding protein (LBP) in PD patients, indicating enhanced exposure to bacterial endotoxins [146]. Such gut-derived factors may propagate neuroinflammation and contribute to blood–brain barrier dysfunction, reinforcing the concept of PD as a gut–brain axis disorder.

The integrity of the intestinal barrier is critical in preventing systemic and neural complications. Recent investigations by Yang et al. demonstrated that fucoidan exerted protective effects in the ROT-induced PD mouse model. Specifically, fucoidan treatment significantly upregulated the expression of Zo-1 and occludin proteins in the colon, which were notably reduced in PD mice, thereby restoring intestinal barrier integrity and ameliorating gut dysfunction. These findings highlight the importance of intestinal barrier maintenance in halting disease progression. Furthermore, intestinal barrier breakdown leads to the translocation of microbial metabolites and bacterial products, which elicit systemic immune activation and central neuroinflammation, associated with several brain disorders, including AD [154,155], depression [156,157], and cognitive dysfunction [158,159]. The restoration of intestinal barrier function may therefore represent a promising intervention target not only in PD but also across multiple neurological disorders.

#### 2.3.3. Mechanistic Insights

Fucoidan conferred protection against PD by modulating the microbiota–gut–brain axis. It induced a microbial remodeling characterized by a reduction in *A. muciniphila* and *L. johnsonii* and an increase in *L. murinus*. This shift in microbiota composition facilitated the restoration of the damaged intestinal barrier. Consequently, the translocation of bacterial LPS and pro-inflammatory cytokines, including TNF-α and IL-1β, into the systemic circulation was significantly reduced. The mitigation of systemic inflammation was further mediated by the downregulation of the LPS/TLR4/NF-κB signaling pathway. By limiting the entry of gut-derived inflammatory mediators into the brain, fucoidan subsequently inhibited microglial activation and attenuated neuroinflammation, ultimately preserving dopamine neurons from damage [25]. The causal role of these specific microbial changes in mediating fucoidan’s benefits was subsequently confirmed through FMT and strain supplementation studies, as detailed in Section 3.3.

### 2.4. Integration Between Gut-Organ Axes

A comparative analysis of the studies presented in the previous sections revealed that the therapeutic benefits of fucoidan in disparate extraintestinal diseases were supported by a set of shared mechanisms. These common pathways, which operated through the gut–organ axes, originated from the modulation of the gut microbial ecosystem. This modulation led to a consequent reinforcement of barrier integrity and the attenuation of systemic inflammation and oxidative stress. This section provides an integrated overview of these unifying principles, with key experimental parameters and repetitive outcomes summarized concisely in Table 1 and Table 2 to facilitate cross-study comparison.

#### 2.4.1. Shared Mechanistic Patterns

**Figure 2:**
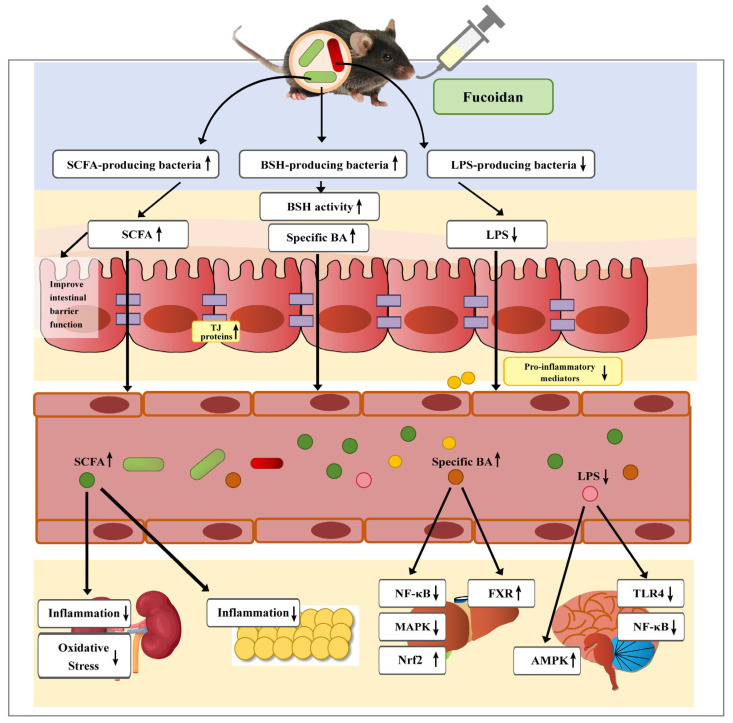
Mechanistic patterns of fucoidan in treating extraintestinal diseases via the gut–organ axis.

Across the gut–brain, gut–liver, gut–kidney, and gut–endocrine axes, a consistent pattern emerged in which fucoidan ameliorated pathology through coordinated biological processes (Figure 2). The compound demonstrated a capacity to remodel the gut microbiota by enriching beneficial commensal bacteria, particularly SCFA producers, while suppressing potential pathogens. This microbial restructuring was closely associated with elevated concentrations of SCFAs in the gut lumen, where these metabolites functioned as critical signaling molecules. Beyond reinforcing the intestinal epithelial barrier, SCFAs entering the systemic circulation exerted broad anti-inflammatory and antioxidant effects throughout the body [28,58,79,160].

Additional key mechanisms involved the modulation of other microbial metabolites, particularly BAs and LPS. Fucoidan administration consistently regulated BA metabolism by altering microbial bile salt hydrolase (BSH) activity and shifting BA composition, subsequently activating FXR signaling pathways in the liver [24,59]. Simultaneously, fucoidan reduced circulating LPS levels by enhancing intestinal barrier integrity and suppressing LPS-producing bacteria, thereby decreasing activation of the TLR4/NF-κB signaling pathway [28,161].

The anti-inflammatory activity of fucoidan represented another central mechanism, with administration consistently leading to downregulation of key pro-inflammatory cytokines including TNF-α, IL-6, and IL-1β, alongside inhibition of NF-κB and MAPK signaling pathways across multiple organ systems [59,79,100,161]. This anti-inflammatory effect was frequently accompanied by reduced oxidative stress, manifested through enhanced antioxidant capacity and diminished lipid peroxidation. Complementing these systemic benefits, fucoidan contributed to barrier integrity restoration at both intestinal and organ-specific levels, thereby preventing translocation of inflammatory mediators and bacterial products while disrupting a fundamental driver of chronic disease progression [28,58,160,161].

#### 2.4.2. Comparative Analysis of Experimental Studies

Building upon these shared mechanistic patterns, a systematic comparison of experimental designs further revealed both consistent approaches and informative variations across studies (Table 2). The effective oral dosage of fucoidan typically ranged from 100 to 300 mg/kg/day, with some disease models showing therapeutic effects at lower concentrations. Treatment protocols were aligned with pathological timelines, progressing from 28-day interventions in acute injury models such as alcohol-induced liver damage to 24-week regimens in chronic conditions, including DKD and HFD-induced obesity [58,79,160]. This strategic alignment reflected the progressive nature of the targeted pathologies while ensuring adequate exposure duration for meaningful therapeutic evaluation.

Comparative analysis demonstrated that while pathological manifestations were organ-specific, fucoidan’s therapeutic mechanisms converged through common upstream pathways. Although different disease models exhibited distinct clinical endpoints, studies consistently confirmed that microbial restructuring, enhanced SCFA production, and subsequent reduction in inflammation and oxidative stress constituted the core protective mechanisms. This mechanistic consistency indicated that fucoidan modulated fundamental physiological pathways to generate trans-organ protective effects, thereby establishing a solid theoretical foundation for its investigation in other pathological conditions involving gut microbiota dysregulation.

#### 2.4.3. Evidence Limitations and Methodological Considerations

Building upon these shared mechanistic patterns, a systematic evaluation of the available evidence reveals several methodological constraints that require attention. Current knowledge of fucoidan’s therapeutic effects relies predominantly on preclinical investigations, which limit their direct translation to human applications. Nevertheless, recent clinical trials have begun to validate these mechanisms in human populations. For instance, a 12-week randomized controlled trial demonstrated that daily supplementation with 1000 mg fucoidan significantly improved glucose metabolism, reduced systemic inflammation (TNF-α, IL-6, LPS), and modulated gut microbiota (e.g., increased *Megamonas* and *Blautia*, decreased *Klebsiella*) in individuals with prediabetes, supporting the translational potential of fucoidan [162].

Moreover, the clinical relevance of the implemented dosing strategies remains uncertain, as human-equivalent doses derived from the effective animal range (typically 100–300 mg/kg/day) pose significant challenges for practical clinical implementation regarding feasibility and safety. Additional considerations involve the level of mechanistic validation and material characterization. Although the correlation between microbial modifications and physiological improvements is well-established, most studies present associative rather than causal evidence, indicating a need for interventional methodologies to confirm direct mechanistic relationships between specific fucoidan structures, microbiota-mediated effects, and therapeutic outcomes. The research field also faces substantial challenges in standardizing fucoidan preparations, where source variability, sulfate content, and molecular weight differences are often inadequately documented. This lack of standardization complicates reliable cross-study comparisons and impedes the identification of the most bioactive compounds.

To address these limitations and advance the field, future research should prioritize several key directions. Clinical validation is essential to verify the mechanisms observed in preclinical models and establish dose–response relationships in human populations. Concurrently, developing nanodelivery systems represents a promising strategy to overcome fucoidan’s bioavailability limitations and enhance its therapeutic potential. These approaches, combined with improved material characterization and causal experimental designs, will strengthen the evidence base and support successful clinical translation of fucoidan-based interventions.

## 3. Microbiota-Mediated Fucoidan Effects: Evidence from FMT and Strain Supplementation

### 3.1. Gut Microbiota Remodeling via FMT and Strain Supplementation

Given the established impact of gut dysbiosis on predisposition to extraintestinal diseases, microbiota-based therapies represent promising therapeutic avenues. These approaches encompass three primary strategies: direct, indirect, and alternative microbiota modulation [33]. Indirect modulation involves interventions such as dietary adjustments [163,164], environmental modifications [160,165], and pharmacological treatments [166,167]. Direct modulation entails strain depletion (e.g., via targeted antibiotics [168,169]) or strain supplementation (e.g., with probiotics [170,171] or defined bacterial isolates [172,173]). Alternative strategies include FMT, which effectively replaces endogenous microbiota via engraftment of donor-derived microbial communities, demonstrating in both animal experiments and clinical applications [174,175].

Clinical and experimental data demonstrated that defined bacterial strains reconfigured gut microbial communities, consequently alleviating extraintestinal disorders via host-microbiota crosstalk. In a 12-week trial of patients with low baseline *Akkermansia muciniphila*, AKK-WST01 supplementation achieved effective gut colonization and significantly lowered HbA1c in overweight/obese type 2 diabetes [176]. Jiang et al. identified depleted probiotic strains (e.g., *Lactobacillus*, *Bifidobacterium*) in painful diabetic neuropathy (PDN) models. Multi-strain probiotic intervention restored gut ecosystem integrity by repairing intestinal barrier function and reducing systemic endotoxemia. This microbiota-directed approach consequently alleviated neuropathic pain and suppressed neuroinflammation [171]. Collectively, these microbiota-directed interventions constituted evidence for microbiota-based precision therapy.

Increasing investigations demonstrated that FMT induced therapeutic restructuring of gut microbial communities, achieving sustained displacement of dysbiotic microbiota in extraintestinal diseases through successful colonization of donor-derived communities. For instance, experimental evidence confirmed that transplanting fecal microbiota from healthy donors or murine models into diseased hosts restructured gut microbial communities. Fan et al. administered encapsulated FMT from healthy donors to hypertension patients, modulating gut microbiota with observed alterations in microbial richness and community structure [177]. Wang et al. employed FMT from healthy female donors to reconstitute the gut microbial community in MCAO mice. Their results demonstrated that transplantation of female-derived microbiota significantly attenuated systemic levels of pro-inflammatory cytokines after ischemic stroke [34]. Furthermore, fecal matter from rodents subjected to experimental interventions is transplanted into intervention-free rodents to accomplish microbial transfer in several studies. Evidence established Fengshining-mediated restoration of gut microenvironment homeostasis as a therapeutic strategy. Mechanistically, FMT from Fengshining-treated donors drove a structural reorganization of the gut microbiota, which effectively ameliorated both microbial dysbiosis and rheumatoid arthritis manifestations [35]. Additionally, human-derived fecal microbiota is transplanted into rodent recipients in certain studies to establish stable microbial colonization. Wang et al. demonstrated that FMT from HCC patients reconstituted a dysbiotic gut microbiota in recipient mice, marked by pathobiont colonization. Metagenomic analyses and bacterial culturing further confirmed the specific enrichment and translocation of the gut pathobiont *Klebsiella pneumoniae* in both HCC patients and FMT-receiving mice [36]. Notably, FMT from clinically distinct donor groups resulted in divergent gut colonization profiles in recipient mice. For instance, transplantation from donors with post-stroke cognitive impairment (PSCI) into stroke model mice induced detrimental microbial remodeling compared to FMT from non-PSCI (nPSCI) donors. This dysbiotic state was characterized by enrichment of *Enterobacteriaceae*, reduction in butyrate-producing taxa, elevated intestinal TLR4 expression, and impaired intestinal barrier integrity [37].

### 3.2. Experimental Workflow of FMT

FMT: (1) Donor screening: Donor screening strategies are customized to align with specific research objectives. FMT serves as a key tool to establish the causal role of gut microbiota in disease modification and therapy. For instance, Jiang et al. used encapsulated FMT from healthy donors to significantly improve autonomic dysfunction in PD patients [178]. Similarly, transplantation of healthy donor microbiota ameliorated myocardial injury in experimental autoimmune myocarditis mice [179]. Furthermore, FMT elucidates the gut microbiota’s mediation of pharmacological efficacy. Geng et al. demonstrated that FMT from inulin-treated PCOS patients alleviated metabolic and ovarian dysfunction in recipient mice [180]. Likewise, Wang et al. confirmed that FMT mediated the anti-obesity effects of the Shengmai San formula [181]. Transplanting human donor microbiota into animal models also helps establish causal relationships by recapitulating disease phenotypes [182].

(2) Recipient conditioning: Several animal models are commonly used as FMT recipients, including conventional, antibiotic-pretreated, GF, vertically transmitted, laxative-preconditioned, and co-housed models. The selection depends on the research goals. GF animals are ideal for mechanistic studies due to the absence of pre-existing microbial competitors, which facilitates exogenous microbiota engraftment. For example, Huang et al. colonized GF mice with PCOS patient microbiota, successfully recapitulating disease-specific dysfunction [183]. Antibiotic-pretreated models offer a practical alternative, as they are cost-efficient and avoid the specialized housing needed for GF animals. This model has been used to confirm microbiota-driven pathogenesis in diseases like pre-eclampsia [123].

Fecal sample processing: This process includes collection, storage, and microbial suspension preparation. ① Collection: As colonic microbiota are predominantly obligate anaerobes, fecal samples must be handled under anaerobic conditions [184]. Donor mice are typically housed in sterilized cages for spontaneous defecation [185], or gentle abdominal palpation may be applied [35,186]. Freshly excreted pellets are collected using sterile instruments and promptly transferred into anaerobic conditions for processing. ② Storage: To preserve microbial integrity, samples for processing within 24 h can be stored at 4 °C. For long-term storage, immediate freezing at −80 °C is recommended, as bacterial community structure remains stable for up to two years. General preparation steps include homogenizing feces in saline (PBS), removing particulate debris, and using the supernatant for transplantation [187,188]. ③ Microbial suspension preparation: Fresh fecal samples are typically homogenized in sterile, oxygen-depleted PBS at ratios ranging from 1:3 to 1:6 (*w*/*v*). Cryopreserved aliquots are thawed in a 37 °C water bath, often with additives like L-cysteine hydrochloride to maintain anaerobic integrity. Processed suspensions are usually filtered and centrifuged to isolate microbial fractions and should be transplanted within a short timeframe post-preparation [189]. In clinical practice, FMT is administered via upper or lower gastrointestinal routes. For rodent models, intragastric gavage is the preferred method due to its simplicity and precise dosage control [190].

### 3.3. Application of FMT and Strain Supplementation in Fucoidan Mechanism Validation

#### 3.3.1. FMT Validation: Gut Microbiota as Essential Mediator

**Figure 3:**
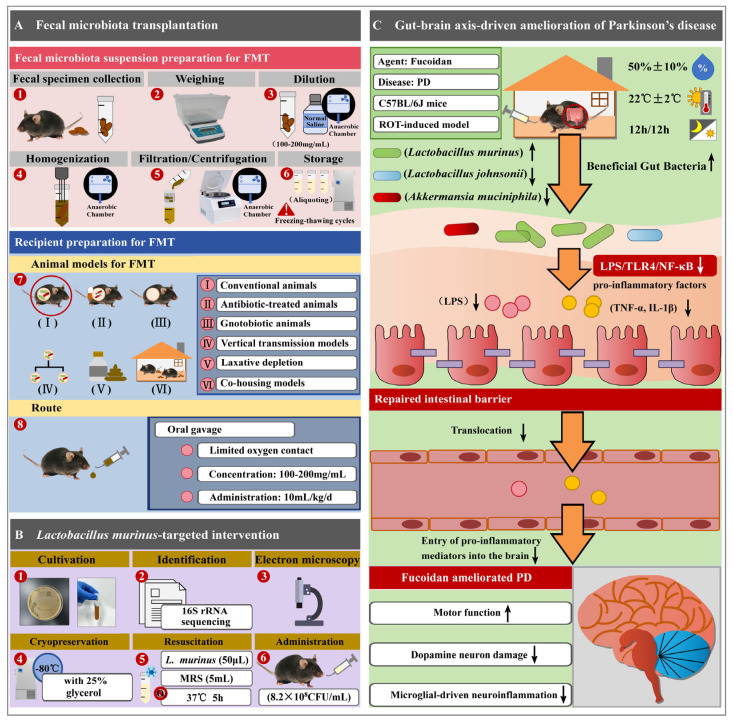
Establishing microbiota–gut–organ axes as therapeutic targets for fucoidan. Evidence from FMT and precision microbiota interventions. (**A**) FMT; (**B**) *Lactobacillus murinus*-targeted intervention; (**C**) gut–brain axis-driven amelioration of PD.

FMT and defined strain supplementation serve as experimental tools to investigate how gut microbiota modulates the therapeutic efficacy of pharmaceutical agents against extraintestinal diseases. Using PD research on fucoidan as a representative example [25], we detailed how FMT and defined strain supplementation validate fucoidan’s mechanistic pathways, particularly its microbiota-mediated neuroprotective effects (Figure 3).

In ROT-induced PD mice, FMT from fucoidan-treated donors (ROT + FH group) fully transferred therapeutic benefits, including attenuated dopaminergic neuron loss, improved motor coordination, and suppressed neuroinflammation. Critically, recipient mice exhibited synchronized microbial remodeling characterized by depletion of mucin-degrading *A. muciniphila* and pro-inflammatory *L. johnsonii*, alongside enrichment of barrier-protective *L. murinus*. Notably, whereas prior studies circumvented causal validation by using GF hosts or microbiota-depleted models, Yang et al. established direct evidence in conventional animals with intact resident microbiota through this FMT approach [191]. This microbial shift identified the gut microbiota as the key driver of fucoidan’s systemic neuroprotection. To dissect causal species within this ecological shift, the study progressed from community-level transfer to targeted bacteriotherapy.

#### 3.3.2. Strain-Specific Reconstitution: *L. murinus* as Sufficient Effector

Supplementation with live *L. murinus* recapitulated the full spectrum of neuroprotection mediated by FMT in a PD model, evident through the restoration of dopamine metabolites, attenuation of microgliosis, and repair of intestinal barrier integrity. This functional alignment established *L. murinus* as a pivotal species that ameliorated *A. muciniphila*-driven pathology. Mechanistically, both interventions suppressed gut–brain TLR4/NF-κB signaling while enhancing IL-10 production. By establishing microbial causality, these findings elucidate fucoidan’s mode of action via ecosystem-mediated remodeling of the gut microbiota.

#### 3.3.3. Microbial Gut–Brain Reprogramming by Fucoidan Reverses PD Neurodegeneration

Fucoidan exerted neuroprotective effects in ROT-induced PD mice by reprogramming gut microbial communities through dual mechanisms: enriching the barrier-protective *L. murinus* while suppressing pro-inflammatory taxa (*A. muciniphila* and *L. johnsonii*). This microbiota remodeling restored intestinal barrier integrity, counteracted *A. muciniphila*-mediated mucus degradation, and reduced endotoxemia (serum LPS). Consequently, translocation of luminal LPS and pro-inflammatory cytokines (TNF-α, IL-1β) into systemic circulation was diminished, attenuating peripheral inflammation.

The resealed intestinal barrier and reduced endotoxemia collectively suppressed TLR4-driven neuroinflammation through downregulation of the LPS/TLR4/NF-κB signaling pathway. This limited brain exposure to neurotoxic mediators, inhibited microglial activation, and ultimately protected dopaminergic neurons from neuroinflammation-induced degeneration. Collectively, this establishes fucoidan as a pioneer in microbial ecosystem engineering for brain disorders, wherein probiotic enrichment and pathobiont inhibition synergistically preserve neuronal homeostasis.

## 4. Conclusions

In this review, we systematically explored the role of fucoidan in treating extraintestinal diseases through the microbiota–gut–organ axes. Our conclusions are organized into three key aspects: (1) Microbiota–gut–organ axes dysfunction contributes to extraintestinal disease pathogenesis, as alterations in gut microbial composition, abundance, and ecological networks are closely linked to the development of extraintestinal disorders. Gut-derived metabolites, such as SCFAs and LPS, play dual roles in organ communication, with their effects determined by concentration and context. Furthermore, intestinal barrier integrity is crucial for limiting microbial translocation, maintaining immune homeostasis, and preventing systemic inflammation. (2) Microbial transfer experiments validated causal mechanisms of fucoidan. A significant challenge in the field has been establishing causative links between microbial changes and disease improvement. FMT and targeted bacterial supplementation have emerged as essential methods to overcome this obstacle. By transferring microbiota from fucoidan-treated donors, studies demonstrated that microbial remodeling is sufficient to elicit the therapeutic benefits, thereby establishing a causal contribution of gut bacteria to the efficacy of fucoidan. (3) Fucoidan acts through regulation of the microbiota–gut–organ axes. FMT-based studies revealed that fucoidan promoted beneficial bacteria, suppressed pathogens, modulated microbial metabolites, and enhanced intestinal barrier function. This leads to the amelioration of organ damage by targeting key processes, including mitigating oxidative stress and suppressing inflammatory responses.

Based on current evidence and existing limitations, future research should focus on three main directions: (1) Clinical validation of fucoidan-mediated mechanisms, as well-designed human trials are needed to verify the microbiota-dependent mechanisms observed in preclinical studies and evaluate the efficacy of different fucoidan types. (2) Standardization of fucoidan preparations, as addressing challenges related to source variability, sulfate content, and molecular weight, is crucial for ensuring reproducible results and identifying the most bioactive compounds. (3) Development of nanodelivery systems, because exploring nanoformulations offers promising approaches to overcome fucoidan’s bioavailability limitations and enhance its therapeutic potential. These research priorities, combined with advanced microbiota intervention techniques, will accelerate the clinical translation of fucoidan-based therapies for extraintestinal diseases.

## Figures and Tables

**Table 1:** Evidence that fucoidan modifies extraintestinal diseases via the microbiota–gut–organ axis.

Gut–Organ Axis	Source of Fucoidan	Disease	Murine Strain	Model	Dose	Crosstalk Among the Microbiota, Gut, and Organ	Potential Mechanism	References
Gut–brain axis	*Saccharina japonica*	Parkinson’s disease	Male C57BL/6J mice	ROT-induced PD model	The low, medium, and high fucoidan treatment groups were 50 mg/kg/d, 100 mg/kg/d, and 200 mg/kg/d, respectively. (Oral gavage, once a day for 21 days)	(1) Correlation: ① Positive correlation: motor function and THir neuron number with intestinal function, intestinal barrier integrity, and *L. murinus*. ② Negative correlation: motor function and THir neuron number with *A. muciniphila*, *L. johnsonii*. (2) Modulation of microbiota (FMT or *L. murinus* intervention): ① Gut: intestinal function ↑, intestinal barrier ↑, mucosal damage, and inflammation ↓. ② Brain: motor function ↑, dopamine neuron damage ↓, and neuroinflammation ↓.	The improvement of PD by fucoidan was attributed to the regulation of the gut microbial ecosystem and the down-regulation of LPS/TLR4/NF-κB signaling pathway	Yang et al. [29]
Gut–brain axis	*Saccharina japonica*	Cognitive dysfunction	Male Sprague Dawley rat	D-gal-induced cognitive dysfunction model	The low and high fucoidan treatment groups were 100 mg/kg/d and 200 mg/kg/d, respectively. (oral gavage, once a day for 8 weeks)	(1) Modulated the gut microbiota: ① Altered microbial community structure. ② Regulated the key bacteria (*Firmicutes* ↓, *Bacteroidota* ↑, and *Akkermansia* ↑). ③ Correlation: a. Positive correlation: brain TNF-α, IL-6, IL-1β, MDA and serum LPS with *Firmicutes*. b. Negative correlation: brain IL-1β with *Bacteroidota*. (2) regulated APN-AMPK-SIRT1 signaling pathway: enhanced the expression of p-AMPKα (Thr172)/AMPKα ratio, APN, and SIRT1 in the hippocampus. (3) Improved mitochondrial dysfunction: increased the expression of PGC-1α, NRF1, and TFAM in the hippocampus. (4) Ameliorated cognitive dysfunction: learning and memory abilities ↑, and histopathological changes ↓.	Fucoidan could reduce oxidative stress and inflammation levels, improve mitochondrial dysfunction to ameliorate cognitive dysfunction by regulating APN-AMPK-SIRT1 signaling pathway, and the gut microbial ecosystem	Xu et al. [30]
Gut–liver axis	*Scytosiphon lomentaria*	Alcoholic liver disease	Male BALB/c mice	Alcohol-induced liver injury model	100 mg/kg/d. (oral gavage, once a day for 60 days)	(1) Modulated the gut microbiota: ① Microbiota diversity ↑. ② Regulated the key bacteria (*Proteobacteria* ↓, *Bacteroidetes* ↓, *Parabacteroides* ↑, *Bacteroides* ↑, and *Faecalibaculum* ↑). (2) Improved liver injury via BA-FXR pathway: ① Regulated the microbial metabolite (BA) in the gut: T-MCA ↓, TUDCA ↓, GLCA ↓, UDCA↓, LCA ↑, and DCA ↑. ② Improved expression of BA-associated parameters (FXR ↑, SHP ↑, Cyp7a1 ↓, Cyp27a1 ↓) in the liver.	Low Mw fucoidan had the potential for the management of ALD by regulating the gut microbiota-BA-liver axis	Sun et al. [23]
Gut–liver axis	*Apostichopus japonicus*	Alcoholic liver disease	Male BALB/c mice	Alcohol-induced liver injury model	The low and high fucoidan treatment groups were 100 mg/kg/d and 300 mg/kg/d, respectively. (oral gavage, once a day for 28 days)	(1) Modulated the gut microbial ecosystem: ① Regulated the key gut microbiota: a. Improved the pro-inflammatory bacteria (*Clostridia_UCG-014* ↓). b. Improved the SCFA-producing bacteria (*Muribaculaceae* ↑ and *Lactobacillaceae* ↑). ② Regulated the microbial metabolites: SCFA concentrations (acetic acid ↑, butyric acid ↑) in the cecal contents. ③ Improved the intestinal barrier: enhanced the expression of TJ proteins (Zo-1 ↑ and Occludin ↑). (2) Exerted protective effects on ALD mice: ① Improved liver function (serum TC ↓, LDL-C ↓, HDL-C ↑) ② Regulated liver oxidative stress levels (antioxidant enzyme activity ↓, GSH ↓). ③ Alleviated the pathological damage of the liver and ileum tissues.	Fucoidan improved alcoholic liver disease based on the synergistic effect of repairing the intestinal mucosal barrier, enhancing the production of SCFAs, and regulating the composition of gut microbiota.	Li et al. [31]
Gut–liver axis	*Scytosiphon lomentaria*	Alcoholic liver disease	Male BALB/c mice	Alcohol-induced liver injury model	300 mg/kg/d. (oral gavage, once a day for 50 days)	(1) Modulated the key gut microbiota (*Parabacteroides distasonis* ↑). (2) Microbial *(Parabacteroides distasonis*) interventions: ① Gut: a. The improved gut microbiota was negatively correlated with indicators of liver injury (ALT, AST, LDL-C, MDA, TC, TG). b. Improved dysbiosis of the BAs profile (BSH activity ↑, TCA ↓, T-MCA ↓, UDCA ↓, DCA ↑, CA ↑, HCA ↑, HDCA ↑). ② Liver: a. Liver index ↓, liver function parameters (serum AST, ALT, TC, TG, and liver LDL-C) ↓. b. Inflammatory infiltration ↓, the cytokines (IL-6 and TNF-α) ↓. c. The activation of the NF-κB and MPAK pathways ↓, the activation of Nrf2/HO-1 ↑. d. Improved expression of BA-associated genes (*Cyp7a1* ↓, *Cyp27a1* ↓, *SHP* ↑).	Fucoidan held great potential to alleviate ALD via *Parabacteroides distasonis*-mediated regulation of the gut-liver axis	Wang et al. [32]
Gut–kidney axis	*Undaria pinnatifida*	Diabetic kidney disease	Male C57BL/6J mice	24 Week HFD-induced DKD model	The low, medium, and high fucoidan treatment groups were 50 mg/kg/d, 100 mg/kg/d, and 200 mg/kg/d, respectively. (oral gavage, once a day for 24 weeks)	(1) Modulated the gut microbial ecosystem: ① Modulated the gut microbiota: SCFA-producing bacteria ↑. ② Regulated the microbial metabolites: SCFAs (acetate ↑, ethylsuccinic ↑) in cecal contents. (2) Improved mitochondrial function: (MDA ↓, SOD ↑, CAT l ↑). (3) Improved inflammation via the MAPKs signaling pathway: alleviated inflammation (IL-6 ↓, IL-1β ↓, IL-10 ↑). (4) Ameliorated DKD. ① Kidney function (urine creatinine ↓). ② Renal histopathology (glomerular hypertrophy, collagen deposition, interstitial fibrosis score, the positive area of TGF-β1 and Col-1) ↓.	Fucoidan improved DKD by promoting acetic acid production, improving mitochondrial function, and inhibiting the MAPKs signaling pathway.	Zhong et al. [33]
Gut–kidney axis	*Saccharina japonica*	Uric acid-induced kidney injury	Male C57BL/6J mice	Potassium oxonate and adenine-induced model	The low, medium, and high fucoidan treatment groups were 150 mg/kg, 200 mg/kg, and 300 mg/kg, respectively. (Oral gavage, once a day for 10 weeks)	(1) Modulated the gut microbial ecosystem: ① Regulated the key gut microbiota: SCFA-producing bacteria ↑. ② Regulated the microbial metabolites: SCFA concentrations (butyric acid ↑, acetic acid ↑, and valeric acid ↑) in intestinal contents. ③ Improved the intestinal barrier (LPS ↓, DAO ↓, permeability ↓). (2) Activated the AMPK/AKT/CREB pathway: regulated uric acid transporters and pathway (ABCG2↑, AMPK/AKT/CREB ↑). (3) Ameliorated kidney injury: ① Kidney function (kidney index ↑, serum uric acid, and creatinine ↓). ② Renal histopathology (glomerular and tubular damage ↓). ③ Improved renal inflammation factors (TNF-α ↓, IL-18 ↓, IL-6 ↓, and IL-1β ↓). ④ Regulated the expression of protein (p-NF-κB p65 ↓, NLRP3 ↓, cleaved caspase-1 ↓, and IL-1β ↓). ⑤ Regulated renal cell apoptosis (BAX ↓ and BCL-2 ↑). ⑥ Regulated renal urate-related transporters (URAT1 ↓ and GLUT9 ↓).	Fucoidan improved the gut microbial ecosystem and activated the AMPK/AKT/CREB pathway in the small intestine to up-regulate the expression of ABCG2, thereby promoting the excretion of uric acid.	Xue et al. [27]
Gut–endocrine axis	*Sargassum fusiforme*	Type 2 Diabetes	Male ICR mice	HFD/STZ-induced T2D model	100 mg/kg/day. (oral gavage, once a day for four weeks)	(1) Modulated the gut microbial ecosystem: ① Regulated the beneficial bacteria (*Bacteroides* ↑, *Faecalibacterium* ↑, and *Blautia* ↑). ② Altered the colonic metabolites (carnitine ↑, choline ↑). (2) Ameliorated T2D: ① Serum lipid ↓. ② Pathological damage of adipose tissue, liver, and heart ↓. ③ Oxidative stress ↓.	Fucoidan improved T2D by improving gastrointestinal health	Wu et al. [34]
Gut–endocrine axis	*Saccharina japonica*	Type 2 Diabetes	Male C57BL/6J mice	Streptozocin-induced T2D model	The low and high fucoidan treatment groups were 150 mg/kg/d and 500 mg/kg/d, respectively. (oral gavage, once a day for 10 weeks)	(1) Modulated the key gut microbiota: SCFA-producing bacteria (*Lactobacillus* ↑, *Allobaculum*). (2) Regulated the microbial metabolites: SCFA concentrations (acetic acid ↑, valeric acid ↑) in the colon. (3) Improved microbial metabolites: amino acids, glutathione, glyoxylate, and dicarboxylate metabolism pathways. (4) Ameliorated T2D: improved glucose and lipid metabolism, oxidative stress, and pancreatic islet integrity.	Fucoidan improved T2D based on regulating gut microbiota and microbial metabolites.	Zhang et al. [35]
Gut–endocrine axis	*Saccharina japonica*	Obesity	Male C57BL/6J mice	HFD-induced obesity model	300 mg/kg/d. (oral gavage, once a day for 8 weeks)	(1) Modulation of microbiota (*Bacteroidota* ↑, *Muribaculaceae* ↑, *Bacteroidaceae* ↑). (2) Improved SCFA generation. (3) Anti-obesity effects: ① The body weight gain ↓, adiposity index ↓, and fat accumulation ↓. ② The serum lipid levels ↓, hypertrophy of adipocytes, and hepatic tissues ↓.	Anti-obesity effects of fucoidan via gut microbiota	Zhang et al. [22]
Gut–endocrine axis	*Sargassum fusiforme*	Obesity	Female C57BL/6J mice	high-fat and high-fructose (HFHF) diet-induced obesity model	0.8 mg/mL/d in the drinking water. (oral administration, once a day for 16 weeks)	(1) Ameliorated obesity-related metabolic disorders: ① Visceral fat accumulation ↓, hyperglycemia ↓, hyperlipidemia ↓. ② BAT ↑, iWAT ↓. (2) Regulated the gut microbiota: the diversity ↑ and the structure of the gut microbiota ↑. (3) Modulation of the gut microbiota (depletion by antibiotic treatment): ① Body weight ↑, body weight gain ↑, BMI ↑, multiple adipose tissue and liver weights ↑, and serum TG ↑. ② Insulin sensitivity ↓, BAT ↓, iWAT ↑.	Fucoidan exerted weight loss and hypolipidemic effects based on increased energy expenditure and reshaped gut microbiota	Zuo et al. [36]
Gut–endocrine axis	*Saccharina japonica*	Obesity	Male C57BL/6J mice	HFD-induced obesity model	fucoidan treatment groups were 100 mg/kg/d, 300 mg/kg/d, respectively. (oral administration, once a day for 24 weeks)	(1) Modulated the gut microbial ecosystem: ① Improved the SCFA-producing bacteria (*Bacteroidetes* ↑ and *Lactobacillus* ↑), and sphingosine-related bacteria (*Erysipelatoclostridium*↓ and *Lachnoclostridium*↓, *Ruminococcaceae_UCG-014* ↓, *Staphylococcus* ↓). ② Regulated the microbial metabolites: fecal SCFA concentrations (acetic acid ↑, propionic acid ↑, isobutyric acid ↑, butyric acid ↑, isovaleric acid ↑, pentanoic acid ↑, and 4-methylpentanoic acid ↑). ③ Improved the intestinal barrier: enhanced the expression of TJ proteins (Zo-1 ↑, Claudin-1 ↑, and Occludin ↑). (2) Improved glucose homeostasis and inflammation: ① Inhibited fat accumulation (Ucp-1 ↑, Prdm16 ↑, Pgc-1α ↑). ② Regulated lipid metabolism (Ppar-α ↑, Ppar-γ ↑, Cpt-1 ↑, Fas↓, Lxr ↓, Srebp-1c ↓). ③ Ameliorated inflammation: (TNF-α ↓, Il-6 ↓, Il-1β ↓, Mcp-1 ↓).	Fucoidan treated obesity through modulation of gut microbiota and lipid metabolites.	Lin et al. [37]

**Table 2:** Targeting shared alterations in the gut microbial ecosystem with fucoidan for extraintestinal diseases.

Target Organ/Disease	Murine	Treatment Protocol (Modeling Induction/Fucoidan Administration)	Common Hallmarks in the Gut Microbial Ecosystem	References
Liver/Alcoholic liver disease	Male BALB/c mice	Alcohol-induced liver injury model; 100 (low-dose) and 300 (high-dose) mg/kg/d (oral gavage, once a day for 28 days).	↑ SCFA-producing bacteria ① *Muribaculaceae* ↑ ② *Lactobacillaceae* ↑ ↑ SCFA concentration in the cecal contents ① Acetic acid ↑ ② Butyric acid ↑ ↑ TJ proteins ① Zo-1 ↑ ② Occludin ↑	Li et al. [31]
Kidney/Diabetic kidney disease	Male C57BL/6J mice	24-week HFD-induced DKD model; 50 (low-dose), 100 (medium-dose), and 200 (high-dose) mg/kg/d (oral gavage, once a day for 24 weeks).	↑ SCFA-producing bacteria ↑ SCFA concentration in the cecal contents ① Acetate ↑ ② Ethylsuccinic ↑	Zhong et al. [33]
Kidney/Uric acid-induced kidney injury	Male C57BL/6J mice	Potassium oxonate and adenine-induced model; 150 (low-dose), 200 (medium-dose), and 300 (high-dose)mg/kg/d (Oral gavage, once a day for 10 weeks).	↑ SCFA-producing bacteria ↑ SCFA concentration in intestinal contents ① Acetic acid ↑ ② Butyric acid ↑ ③ Valeric acid ↑ ↑ Intestinal barrier integrity Permeability ↓	Xue et al. [27]
Endocrine/Obesity	Male C57BL/6J mice	HFD-induced obesity model; 100 (low-dose) and 300 (high-dose) mg/kg/d (oral administration, once a day for 24 weeks).	↑ SCFA-producing bacteria ① *Bacteroidetes* ↑ ② *Lactobacillus* ↑ ↑ Fecal SCFA concentrations ① Acetic acid ↑ ② Butyric acid ↑ ③ Propionic acid ↑ ↑ TJ proteins ① Zo-1 ↑ ② Claudin-1 ③ Occludin ↑	Lin et al. [37]

## Data Availability

Data sharing is not applicable to this article because no data were generated or analyzed during this study.
